# Leprosy in Pregnant Woman, United States

**DOI:** 10.3201/eid1910.130463

**Published:** 2013-10

**Authors:** Alexis C. Gimovsky, Charles J. Macri

**Affiliations:** George Washington University Hospital, Washington, DC, USA

**Keywords:** Hansen disease, pregnancy, leprosy, lepromatous leprosy, *Mycobacterium leprae*, mycobacteria, bacteria, public health

**To the Editor:** Hansen disease, or leprosy, in pregnancy is a rarely reported event in the United States. In 2009, a total of 213,036 new cases of leprosy were detected throughout the world (*1*). Nine countries in Africa, Asia, and Latin America consider it a public health problem, accounting for ≈75% of the global disease prevalence (*1*).

We describe a case of leprosy in a 27-year-old woman with 1 previous pregnancy and 1 live-born infant who had onset of subcutaneous nodules before she became pregnant. She appeared at her initial prenatal visit at 24.1 weeks of gestation after recently emigrating from Mexico. The patient reported that subcutaneous nodules had developed on her arms, legs, back, and abdomen ≈5 months before the visit, 2 weeks before her last menstrual period. A skin biopsy revealed acute and chronic panniculitis with acid-fast bacilli, and the condition was confirmed by PCR to be lepratamatous leprosy. Treatment included rifampin, Dapsone, clofazimine, and prednisone.

The patient’s condition was monitored closely with ultrasounds at serial intervals; these showed consistent fetal growth at the 50th percentile. At 37 weeks and 1 day, her membranes ruptured. She underwent a repeat cesarean delivery because the method of leprosy transmission is not yet proven and to prevent possible vertical transmission to the infant. The patient delivered a female infant weighing 6 lb, 8 oz, with Apgar scores of 8 and 9. On postoperative day 1, Dapsone treatment was restarted; she was given Dapsone, 50 mg daily, and prednisone, 40 mg daily. She was discharged with the baby on postoperative day 3. 

Leprosy is a chronic disease caused by *Mycobacterium leprae*. The disease mainly affects the skin and nerves and, if untreated, can cause permanent damage. It is curable, however, and disability can be avoided. The World Health Organization recommends multidrug therapy consisting of Dapsone, rifampin, and clofazimine (*1*). This combination has proven highly effective, and patients are no longer infectious after the first dose (*1*). Virtually no relapses occur and antimicrobial drug resistance does not develop (*1*). Pregnancy causes a relative decrease in cellular immunity, which allows *M. leprae* to proliferate (*2*). Careful management can prevent permanent nerve damage. Leporatamatous leprosy and relapse after treatment are more commonly seen throughout pregnancy because of the pregnant woman’s immunodeficient state (*2**,**3*).^,^Infants are usually less affected than mothers; nevertheless, selection of the mother's antimicrobial drug regimen must ensure adequate control of the bacteria while avoiding teratogenicity and in utero adverse effects, such as low birthweight (*3*,*4*). The infant has a potentially high risk of contracting leprosy from the mother by skin-to-skin contact or droplet transmission, particularly if she has not received treatment.
